# Biosynthesis of Cry5B-Loaded Sulfur Nanoparticles using *Arthrobotrys oligospora* Filtrate: Effects on Nematicidal Activity, Thermal Stability, and Pathogenicity against *Caenorhabditis elegans*

**DOI:** 10.1021/acsomega.3c08653

**Published:** 2024-02-03

**Authors:** Pasin Jammor, Tanatcha Sanguanphun, Krai Meemon, Boonhiang Promdonkoy, Panadda Boonserm

**Affiliations:** † Institute of Molecular Biosciences, Mahidol University, Salaya, Phuttamonthon, Nakhon Pathom 73170, Thailand; ‡ Department of Anatomy, Faculty of Science, Mahidol University, Rama VI Road, Bangkok 10400, Thailand; § National Center for Genetic Engineering and Biotechnology, National Science and Technology Development Agency, 113 Phahonyothin Road, Khlong Luang, Pathumthani 12120, Thailand

## Abstract

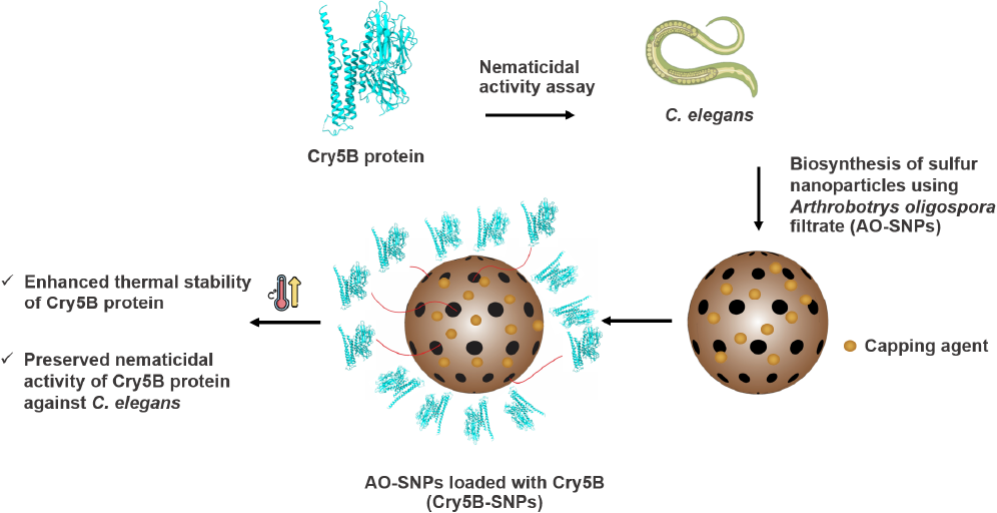

Cry5B, a crystal protein produced by *Bacillus thuringiensis* (Bt), is a bionematicide with potent nematicidal activity against various plant-parasitic and free-living nematodes. This protein, however, is susceptible to destruction by ultraviolet light, proteolytic enzymes, and high temperatures. This study aims to produce Cry5B protein for bionematicidal use and improve its stability and nematicidal efficacy by loading it into*Arthrobotrys oligospora*-mediated sulfur nanoparticles (AO-SNPs). Based on the mortality assay, the Cry5B protein exhibited dose-dependent nematicidal activity against the model organism*Caenorhabditis elegans*. The nematicidal activity, thermal stability, and pathogenic effects of Cry5B-loaded AO-SNPs (Cry5B-SNPs) were compared to those of free Cry5B. After 3 h of exposure to heat at 60 °C, Cry5B-SNPs had greater nematicidal activity than free Cry5B protein, indicating the effective formulation of Cry5B-SNPs that could be used as an alternative to current nematicide delivery strategies.

## 1 Introduction

Plant-parasitic nematodes (PPNs) constitute an important threat to numerous agricultural crops, such as potatoes, soybeans, and tomatoes, accounting for around 15% of total agriculture losses by these pathogens.^1^*Meloidogyne* spp., also known as root-knot nematodes, are the most destructive group of plant parasitic nematodes, causing extensive crop damage by forming large galls or knots throughout the root system of infected plants, which serve as their permanent feeding sites in the root.^2^*Meloidogyne incognita* (*M. incognita*) is among the most economically significant nematodes in tropical and subtropical areas that are difficult to control because they spend most of their lives within plant roots, rendering them resistant to conventional control methods.^3^ Currently, plant-parasitic nematode control is primarily based on chemical nematicides, which have resulted in severe environmental issues and the emergence of PPN strains with chemical resistance. As a result, biological agents, bioactive compounds derived from plants and microorganisms, such as fungi and bacteria, as well as innovative nanoparticle delivery systems for bionematicides, have gained attention for the control of PPNs.

Among the microorganism-based products, the entomopathogenic bacterium *Bacillus thuringiensis* (Bt) has demonstrated excellent insect control, especially against Lepidoptera, Coleoptera, and Diptera. Its insecticidal activity is related to the production of parasporal crystal proteins (Cry) or δ-endotoxins.^4^ Moreover, some Bt strains produce crystal proteins belonging to the families Cry5, Cry6, Cry12, Cry13, Cry14, Cry21, and Cry55 that are nematicidal against both plant and animal nematodes. Out of these proteins, Cry5B is the most extensively studied, characterized, and investigated nematicidal protein in terms of structure and mode of action against multiple nematode species, including free-living, such as *Caenorhabditis elegans*, and plant- and animal-parasitic species.^5,6^ It has been proposed that the nematicidal activity of Cry5B follows a sequence of events similar to those of the insecticidal Cry toxins, including toxin binding to a host receptor, followed by membrane insertion that causes intestinal damage and death of the host.^7^

However, Bt proteins have some limitations for field agricultural applications, such as accelerated denaturation, decreased stability, and bioactivity loss when exposed to high temperatures and continuous sunlight.^8^ In addition, Bt proteins have difficulty in penetrating the soil and plant roots, where PPNs reside. These concerns drive the exploration and development of novel formulations. As a result, the development of nanotechnology for the production of new bionematicide formulations is highly encouraging. Nanoparticles (NPs) are considered effective nanocarriers for nematicide delivery and protect bionematicidal agents from degradation.^9^ Nature-based nanomaterials have been shown to be biocompatible, environmentally safe, and naturally absorbed by plant roots. Sulfur is one of the secondary plant nutrients necessary for healthy growth and development.^10,11^ Because of their low toxicity to humans and wildlife, as well as their rapid dissipation, sulfur compounds are regarded as relatively harmless substances,^12^ making them an attractive candidate to be formulated as sulfur nanoparticles for nematicidal protein delivery and protein protection.

Despite the widespread use of chemical and physical methods for NP synthesis, serious issues in biomedicine and environmental applications have emerged, necessitating the need for safe, reliable, ecofriendly, and cost-effective NP synthesis. In addition, natural products derived from plants, algae, bacteria, and fungi are effective as reducing, stabilizing, and capping agents during nanoparticle synthesis.^13^ For example, filtrates of *Arthrobotrys* spp., *Fusarium* spp., *Aspergillus* spp., and *Penicillium* spp. containing multiple enzymes and metabolites can be used as biological reducing agents to produce either intracellular or extracellular metallic nanoparticles.^14,15^*Arthrobotrys* spp. are a subgroup of nematode-trapping fungi that can capture and digest nematodes using specialized trapping structures and produce a variety of metabolites with nematicidal activity, making them a promising candidate for the control of plant-parasitic nematodes.^16−19^*Arthrobotrys oligospora* is the first identified and by far the most common nematode-trapping fungus in the environment.^20,21^ Interestingly, naturally occurring nanoparticles from *A. oligospora* have been discovered, offering possibilities for biomedical and other applications.^22^

This study aims to develop a simple and environmentally friendly approach for synthesizing extracellular sulfur nanoparticles using a cell-free *A. oligospora* filtrate. The biosynthesized sulfur nanoparticles were loaded with Cry5B, and their ability to protect and deliver the Cry5B protein to target sites was evaluated by measuring their nematicidal activity against the model organism *C. elegans*, with and without exposure to high temperatures. This novel formulation will facilitate future crop protection and chemical pesticide reduction as well as promote sustainable agriculture and food safety.

## 2 Materials and Methods

### 2.1 Bacterial, Fungal, and *C. elegans* Strains

The *B. thuringiensis* (Bt) strain carrying recombinant plasmid BMB171 and containing the *cry5B* gene was kindly provided by Prof. Dr. Ming Sun, Huazhong Agricultural University, China. *A. oligospora* isolate number BCC 3847 was kindly provided by Dr. Alongkorn Amnuaykanjanasin, National Center for Genetic Engineering and Biotechnology, Thailand. Wild-type *C. elegans* N2 strain was used as a model organism in this study, and the *Escherichia coli* OP50 strain was used as a food source for *C. elegans*. All *C. elegans* experiments were ethically performed under the guidelines of the Faculty of Science, Mahidol University–Institutional Animal Care and Use Committee (MUSC–IACUC; MUSC66-001-631).

### 2.2 Cry5B Protein Production and Solubilization

Bt BMB171 strain expressing Cry5B protein was incubated in liquid 2 × SG medium with 25 μg/mL erythromycin for 6 days at 30 °C with shaking at 200 rpm until a crystal form of Cry5B protein was produced, which could be observed under a light microscope. After centrifugation, the Cry5B protein crystals were recovered in a pellet and solubilized in 50 mM carbonate buffer pH 10.5, followed by centrifugation at 12,000 g at 4 °C for 1 h to separate soluble and insoluble fractions. The proteins were analyzed by SDS-PAGE and quantified by using the Bradford assay with bovine serum albumin (BSA) as the standard.

### 2.3 Collection and Preparation of *A. oligospora* Filtrate

*A. oligospora* isolate number BCC 3847 was subcultured when the culture Petri dish of potato dextrose agar (PDA) was covered by all of the fungal mycelium (10 days of cultivation). For subculturing, 0.5 × 0.5 cm^2^ of PDA with fungal mycelium was cut and placed in the middle of a freshly prepared PDA culture Petri dish. The culture Petri dish was then incubated at 25 °C for 10 days in the dark. For fungal filtrate preparation, 4 pieces of 1 × 1 cm^2^ of PDA with fungal mycelium were cut and transferred into the freshly prepared 50 mL potato dextrose broth (PDB) and further incubated at 25 °C for 10 days in the dark to produce secondary metabolites. Finally, a 0.22 μm Millipore filter was used to separate fungal mycelium and obtain the fungal filtrate for further use as a stabilizing or capping agent in nanoparticle synthesis.

### 2.4 Gas Chromatography and Mass Spectrometry (GC-MS) Analysis

After the fungal filtrate of *A. oligospora* was prepared, a gas chromatography–mass spectrophotometer (GC-MS, model 5977B GC/MSD, Agilent, Santa Clara, CA, USA) was used to identify the potential metabolites present in the fungal filtrate. The GC–MS analysis was undergone at the Mahidol University-Frontier Research Facility (MU-FRF), Thailand, following the protocol as described previously.^23^

### 2.5 Preparation of Biosynthesized Sulfur Nanoparticles using *A. oligospora* Fungal Filtrate (AO-SNPs)

Sulfur nanoparticles were prepared following the protocol as described previously.^24^ Briefly, 0.124 g of sodium thiosulfate pentahydrate (STS; Na_2_S_2_O_3_·5H_2_O) was dissolved in 45 mL of *A. oligospora* fungal filtrate. Subsequently, 5 mL of 0.2 M HCl was added dropwise to the solution and continuously stirred until the solution changed from clear to turbid solution (approximately 1 h), which indicates the formation of sulfur nanoparticles (SNPs). Then, the solution mixture was sonicated for 30 min in a bath-type sonicator to complete the reaction. Notably, a 1:2 molar concentration ratio of STS to HCl was maintained throughout the experiment, and STS underwent a transformation into sulfur and sulfonic acid. The pellets containing sulfur nanoparticles were harvested by centrifugation at 8,000 g for 5 min and washed with sterile distilled water 3 times until the pH of the solution became neutral. Subsequently, the suspension was centrifuged at 8,000 rpm for 5 min, and the supernatant was discarded. The pellets containing sulfur nanoparticles (AO-SNPs) were then stored at 4 °C before use. PDB was utilized as a control sample to produce PDB-SNPs by using the same protocol as that for preparing AO-SNPs.

### 2.6 Characterization of Biosynthesized Sulfur Nanoparticles

#### 2.6.1 UV–Visible Spectroscopy

UV–visible spectroscopy was used to preliminarily confirm the formation of nanoparticles. The analysis was carried out in the wavelength region of 190–600 nm by analyzing the light absorption of nanoparticles using a NanoDrop One Microvolume UV–vis Spectrophotometer (Thermo Scientific, Waltham, MA, USA).

#### 2.6.2 Dynamic Light Scattering (DLS) and Static Light Scattering (SLS)

Dynamic light scattering (DLS; SZ-100 particle size analyzer, Horiba, Kyoto, Japan) was used to determine the size distribution of nanoparticles in a liquid sample between 0.3 nm and 8 μm, as well as the surface charge or zeta potential (Z-potential) value of nanoparticles. The size distribution of nanoparticles between 0.1 and 1,000 μm was determined using static light scattering (SLS) or laser diffraction (Particle mini LA-350, Horiba).

#### 2.6.3 Field Emission Scanning Electron Microscopy (FESEM) and Energy Dispersive X-ray Spectroscopy (EDX)

The combination of field emission scanning electron microscopy (FESEM) and energy dispersive X-ray spectroscopy (EDX) is used to analyze morphology features and elemental composition on the surfaces of nanoparticles, respectively. The analysis was performed using FESEM model JSM-7610FPlus, (JEOL, Tokyo, Japan). After synthesizing and harvesting the sulfur nanoparticles, the pellets containing the sulfur nanoparticles were air-dried for 2–4 days or dried in an oven overnight at 60 °C. The dried pellets were mounted on carbon tape on a metal stub. Then, a stub was coated with platinum (Pt), a conductive material, to enhance the conductivity of the samples.

#### 2.6.4 Fourier-Transform Infrared Spectroscopy (FTIR)

Fourier-transform infrared spectroscopy (Nicolet iS50 FTIR Spectrometer, Thermo Scientific, Waltham, MA, USA) was used to identify possible functional groups and various chemical bonds in the samples. After the complete synthesis and collection of sulfur nanoparticles, the pellets containing the sulfur nanoparticles were air-dried for 2–4 days or oven-dried overnight at 60 °C. The pellet sample was prepared by the standard KBr pellet method to perform FTIR and analyzed in the range 400–4000 cm^–1^ using the transmittance mode.

### 2.7 Cry5B-Loaded Sulfur Nanoparticles (Cry5B-SNPs) Preparation

Cry5B-loaded sulfur nanoparticles (Cry5B-SNPs) were prepared by using the precipitation method as described previously.^25^ This process was performed by using a volume ratio of 1:1 of the Cry5B protein and AO-SNPs. Briefly, 3 mg of AO-SNPs in 300 μL of sterile distilled water were mixed with 300 μL of aliquot of Cry5B protein in the solubilization buffer (50 mM carbonate buffer pH 10.5). The mixture was incubated by being placed on a rotator overnight at room temperature. The Cry5B-SNPs were collected by centrifugation at 10,000 rpm for 10 min. The amount of Cry5B loaded into/onto AO-SNPs was determined by subtracting the remaining amount of Cry5B in the supernatant from the total amount of Cry5B added to the sample via the Bradford method with bovine serum albumin (BSA) as the standard. The loading efficacy was calculated as described in a previous study.^26^

### 2.8 Investigation of Nematicidal Activity of Cry5B Protein against *C. elegans*

The nematicidal activity was determined using synchronized L4-stage larvae of*C. elegans*. The synchronized L4-stage larvae were collected and tested in a 48-well plate. Each well contained 125 μL of k-medium, 40 μL of *E. coli* OP50 in the k-medium with absorbance 600 nm = 3.0 (OD_600_ = 3), 5 μL of 8 mM 5-fluoro-2′-deoxyuridine (FUdR), 20 μL of either solubilization buffer (as a control) or the desired Cry5B concentration (in solubilization buffer), and 10 μL of L4-stage larvae in M9 buffer. Then, Cry5B protein at different concentrations was introduced into each well of the 48-well plate containing nematodes and incubated at 25 °C for 5 days. The amount of live *C. elegans* was scored daily under a stereomicroscope and collected as a cumulative viability percentage to determine the LC_50_ concentration value (lethal dose). The living and dead *C. elegans* were distinguished by their responsiveness and motility. *C. elegans* that moved in response to stimulation were labeled as alive, whereas *C. elegans* that did not move in response to stimulation were labeled as dead. The entire assay was repeated three times for each protein concentration. The LC_50_ value was analyzed using IBM SPSS Statistics (version 26 by Probit analysis).

### 2.9 Thermal Stability Assay of Cry5B-SNPs

To examine the thermal stability of Cry5B-SNPs, Cry5B protein was first diluted with a solubilization buffer to obtain a concentration of protein equal to that of Cry5B protein that loaded into/onto SNPs. Then, diluted Cry5B protein (35 μg/mL), Cy5B-SNPs (35 μg/mL of Cry5B protein loaded into/onto SNPs), and free AO-SNPs (as a control) were heated at 60 °C for 3 h. Upon mixing the samples with the constituents in the well, each sample’s final protein concentration was reduced by a factor of 10. The nematicidal activity of each treatment was determined by quantifying the viability of L4-stage larvae of *C. elegans* under each condition as described above.

### 2.10 Propidium Iodide Uptake Assays

Synchronized L4-stage larvae were incubated with unheated Cry5B protein (a final concentration of 3.5 μg/mL), unheated Cry5B-SNPs, unheated AO-SNPs, heated Cry5B protein (60 °C for 3 h), heated Cry5B-SNPs, and heated AO-SNPs. After 5 days of incubation, L4-stage larvae were incubated with 100 μM propidium iodide (Sigma) in the dark for 1 h and then visualized using a fluorescence microscope (Nikon Upright Microscope Eclipse Ci) with the excitation and emission wavelengths of 555 and 580 nm, respectively. The ImageJ software was used to measure the pixel intensity for each nematode.

### 2.11 Statistical Analysis

Statistical analysis (two-way ANOVA) was performed by using GraphPad Prism Version 9. The statistical significance was considered when the *p*-value < 0.05.

## 3 Results and Discussion

### 3.1 Identification of Potential Bioactive Compounds Present in *A. oligospora* Fungal Filtrate

To demonstrate the possibility of biosynthesizing sulfur nanoparticles using *A. oligospora* fungal filtrate, the *A. oligospora* fungal filtrate was subjected to GC-MS analysis to identify potential bioactive compounds that may have acted as reducing, capping, and stabilizing agents for sulfur nanoparticle synthesis. The GC–MS results showed five major volatile organic compounds (VOCs) identified from *A. oligospora* fungal filtrate including (1) benzeneacetaldehyde, (2) 3-ethyl-2,5-dimethyl-pyrazine, (3) phenylethyl alcohol, (4) 2-phenyl-1,3,2-dioxaborolane, and (5) 3,5-bis(1,1-dimethylethyl)-phenol. These compounds are listed in Table 1 based on their retention time (RT) from the HP-5MS column. Most of these VOCs are identified as aldehydes, alcohols, and phenolic compounds. In addition to their role as nanoparticle-mediated compounds, VOCs produced by plants and microorganisms are considered potential substances for the development of nematicides.^27^ For example, the VOCs including benzyl benzoate, benzaldehyde, 2-heptanone, and acetophenone produced by *Bacillus nematocida* B16 showed potent nematode-attracting abilities.^28^ In addition, it has been demonstrated that VOCs produced by fungi possess potent nematicidal properties. According to a previous study, VOCs produced by *Fusarium oxysporum* and *F. solani*, including esters, alcohols, phenols, aldehydes, carboxylic acids, and sesquiterpenes, rendered *M. incognita* J2s immobile *in vitro* and lowered gall and egg production in tomato plants.^29^ In addition, pyrazine, one of the volatile organic compounds identified in the fungal filtrate of *A. oligospora*, was among the metabolites identified in the fungal filtrate of *A. thaumasia* that could be responsible for its nematicidal activity against *M. incognita* and *C. elegans*.^30^ In this study, however, we were unable to observe nematicidal activity when the *A. oligospora* fungal filtrate was applied to *C. elegans* (data not shown), implying that the concentrations of VOCs present in the *A. oligospora* fungal filtrate were insufficient to exert nematicidal activity. Nonetheless, it remained to be determined whether the sulfur nanoparticles mediated by the fungal filtrate of *A. oligospora* (AO-SNPs) could contribute to the nematicidal activity.

**Table 1:** Potential Bioactive Compounds Present in the *A. oligospora* Fungal Filtrate

compound name	RT (min)	formula
benzeneacetaldehyde	9.495	C_8_H_8_O
3-ethyl-2,5-dimethyl-pyrazine	10.704	C_8_H_12_N_2_
phenylethyl alcohol	11.866	C_8_H_10_O
2-phenyl-1,3,2-dioxaborolane	17.283	C_8_H_9_BO_2_
3,5-bis(1,1-dimethylethyl)-phenol	24.989	C_14_H_22_O

### 3.2 Biosynthesis and Characterization of Sulfur Nanoparticles

**Figure 1:**
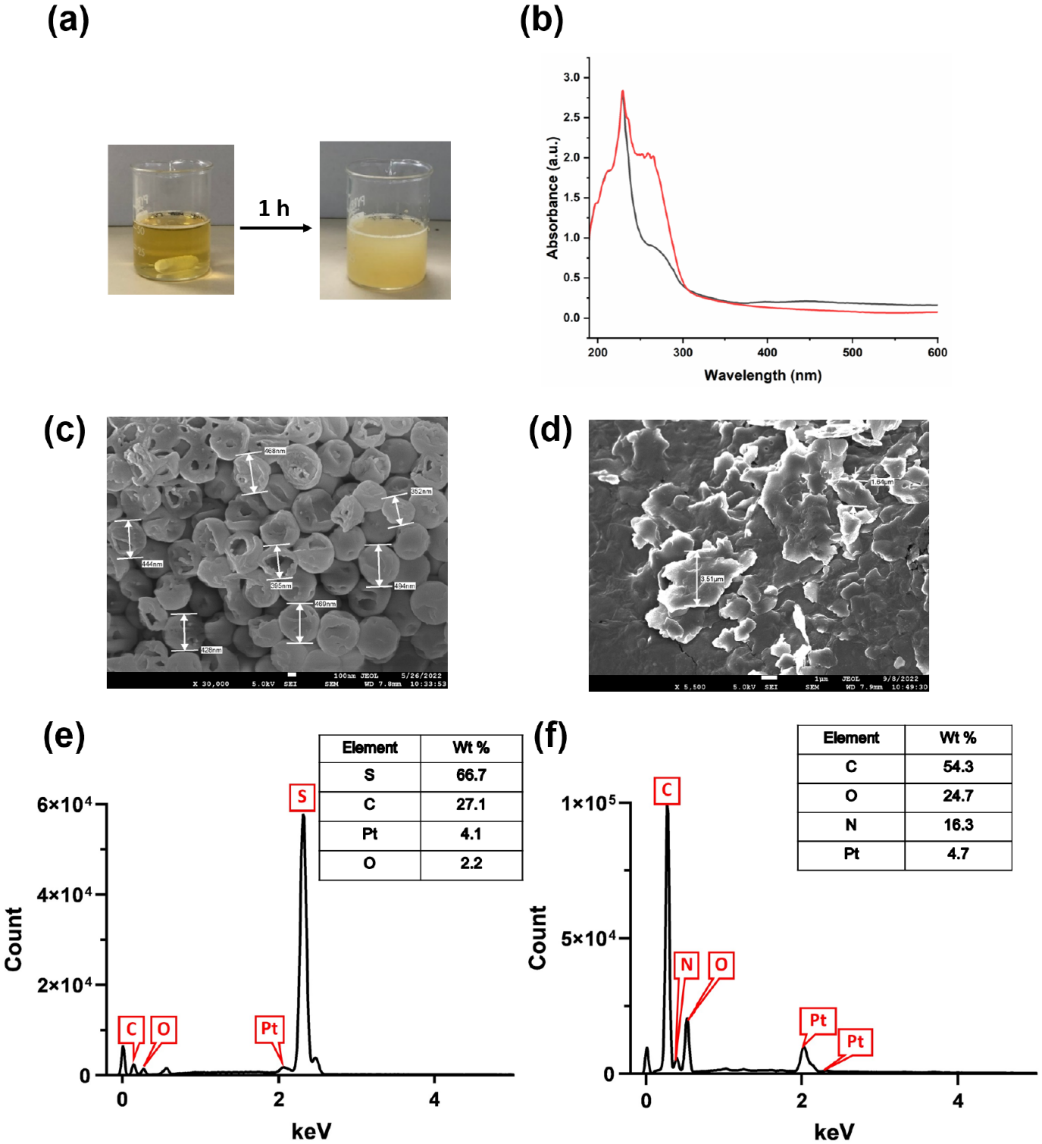
Biosynthesis and characterization of sulfur nanoparticles using *A. oligospora* fungal filtrate (AO-SNPs). Solution’s turbidity changes after the completion of sulfur nanoparticle synthesis (a); UV–Vis spectrum of AO-SNPs (black line) compared with that of PBD-SNPs (red line) (b); FE-SEM analysis of AO-SNPs (c) and PDB-SNPs (d); EDX analysis of AO-SNPs (e) and PDB-SNPs (f).

After mixing *A. oligospora* fungal filtrate with sodium thiosulfate and hydrochloric acid, the formation of sulfur nanoparticles mediated by *A. oligospora* fungal filtrate (AO-SNPs) was first monitored by the appearance of the solution’s turbidity, as the solution’s transition from clear to turbid indicates the formation of nanoparticles (Figure 1a).^31^ As a control, potato dextrose broth (PDB) was used to synthesize SNPs (PDB-SNPs). UV–visible spectroscopy recorded in the wavelength range of 190–600 nm was used to preliminarily confirm the formation of sulfur nanoparticles. It is well-known that α-sulfur exhibits an optical absorption maximum (λ_max_) in the range of 260–280 nm; thus, the presence of absorbance peaks between 260 and 290 nm in the spectra of AO-SNPs and PDB-SNPs indicates the formation of SNPs (Figure 1b).^31,32^

The combination of FESEM and EDX was used to analyze the surface morphology and elemental composition of the biosynthesized SNPs. FESEM images revealed that AO-SNPs had a particle size of approximately 435 nm and a spherical shape with cavities inside the particles (Figure 1c), while PDB-SNPs had a particle size of approximately 2.56 μm with an irregular shape (Figure 1d). To the best of our knowledge, this is the first report on an environmentally friendly method for the synthesis of sulfur nanoparticles using an *A. oligospora* fungal filtrate. The organic compounds employed here have a large influence on the structure and shape of biosynthesized nanoparticles. In this study, porous sulfur nanoparticles were solely found in AO-SNPs and not in PDB-SNPs, indicating that the pores were created by organic compounds present in the*A. oligospora* filtrate. It has been shown that porous hollow silica nanoparticles (PHSNPs) are effective for the controlled release of water-soluble pesticides and for enhancing their transport to specific target sites.^33^ Moreover, the ability of mesoporous silicon particles to protect Cry5B from hydrolytic and enzymatic degradation in simulated gastric fluid (pH 1.2) has been demonstrated.^34^ It was, thus, believed that the porous structure of AO-SNPs would provide significant advantages for high-loading capacity and prevent premature protein release and protein degradation.

The results from EDX analysis showed that sulfur (S) is the major element in AO-SNPs with the highest energy level peak at approximately 2.3 keV (Figure 1e), while carbon (C) is the most abundant element in PDB-SNPs with a peak energy level of approximately 0.3 keV (Figure 1f). These findings suggest that sulfur nanoparticles synthesized by *A. oligospora* fungal filtrate are smaller, more porous, more dispersed, and more uniform than those synthesized without the fungal filtrate. This suggests that extracellular biomolecules present in the fungal filtrate, such as enzymes and metabolites, might act as a capping or stabilizing agent during sulfur nanoparticle biosynthesis and prevent the agglomeration of nanoparticles.

**Figure 2:**
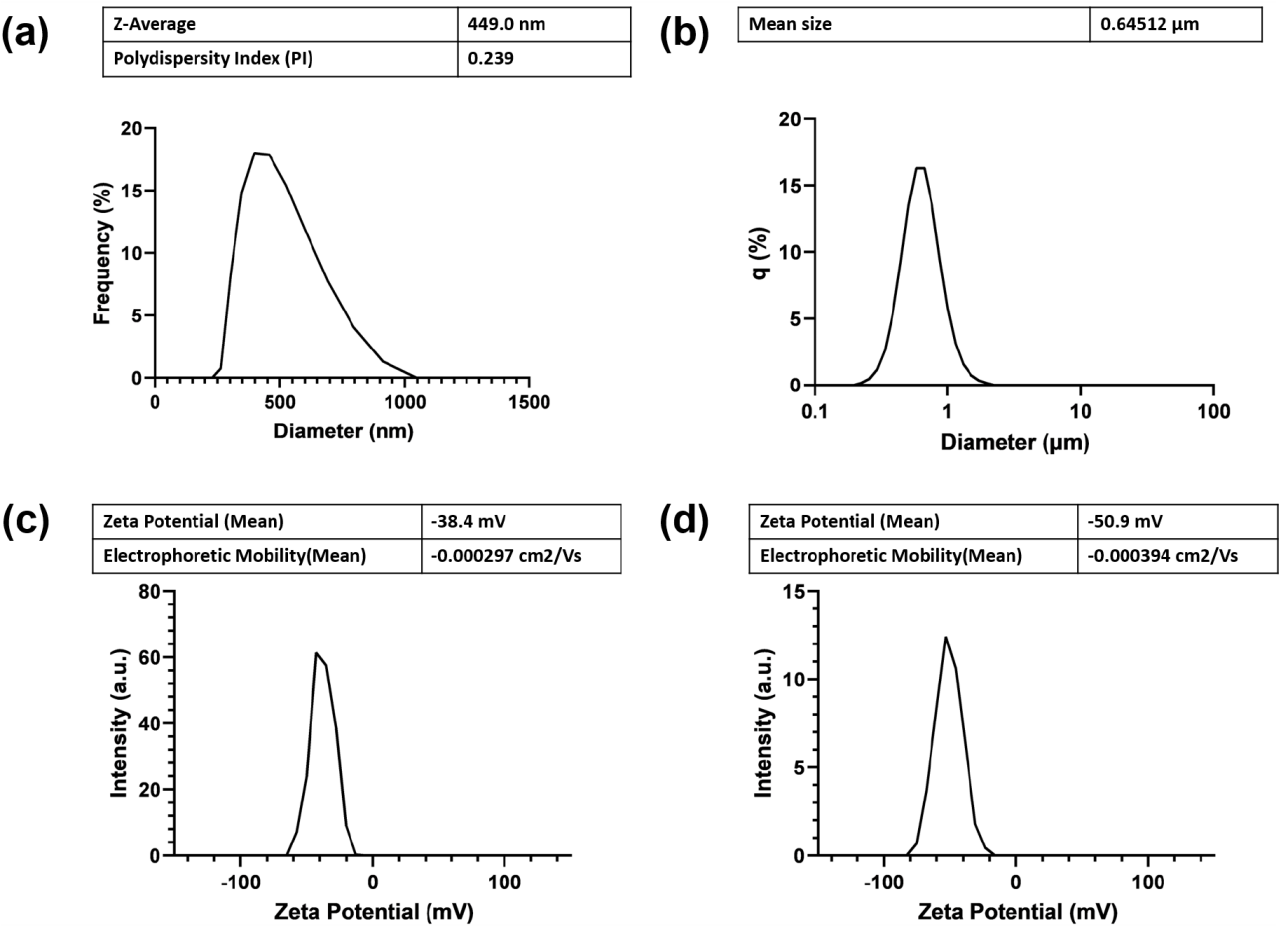
Characterization of biosynthesized SNPs using fungal filtrate (AO-SNPs) compared with SNPs using potato dextrose broth (PBD-SNPs). DLS analysis of AO-SNPs (a) and PDB-SNPs (b); zeta potential analysis of AO-SNPs (c) and PDB-SNPs (d).

Dynamic light scattering (DLS) was also used to determine the size distribution of particle size samples with a submicron scale and provide the surface charge or zeta potential (Z-potential) value of nanoparticles, whereas static light scattering (SLS) or laser diffraction was only utilized to determine the size distribution of nanoparticles with a micron-scale particle size.^35^ The results indicated that the particle size of AO-SNPs, as determined by DLS, had an average size of 450 nm (Figure 2a), which is consistent with the FE-SEM result. In contrast, the average particle size of the PDB-SNPs determined by SLS was 0.6 μm (Figure 2b). DLS is preferable for spherical particle size characterization by measuring the hydrodynamic radius.^36,37^ In contrast to the AO-SNPs, which had a uniform spherical shape, the PDB-SNPs had irregular morphologies; as a result, the hydrodynamic size distribution acquired by using DLS was excessively variable and could not be quantified. However, static light scattering (SLS) is a method for determining the absolute molecular weight by examining the relationship between the light intensity scattered by a particle and its size and molecular weight.^35^ The apparent similarity in particle size values between AO-SNPs and PDB-SNPs measured by SLS could be due to their comparable molecular masses rather than size. This assumption was later supported by an FE-SEM examination of particle size and morphology, which indicated that PDB-SNPs did, in fact, have larger particle sizes. In agreement with the FE-SEM analysis, the AO-SNPs are smaller than the PDB-SNPs, indicating that fungal filtrate serves as an effective capping agent that stabilizes, prevents agglomeration, and regulates the size of SNPs. The surface charge or zeta potential values of the AO-SNPs and PDB-SNPs were −38.4 (Figure 2c) and −50.9 (Figure 2d), respectively. As the zeta potential value of nanoparticles greater than ±30 mV is a key indicator of the stability of a colloidal dispersion, these biosynthesized SNPs were tentatively stable due to the high repulsive force, which decreased the tendency of particles to aggregate.^38^

The FTIR spectra of biosynthesized SNPs were recorded to identify functional groups possibly involved in the reduction of sulfur ions to form sulfur nanoparticles, as shown in Figure S1. PDB-SNPs and AO-SNPs had similar characteristic peaks. Broad absorption peaks at 3443 and 3424 cm^–1^ found in PDB-SNPs and AO-SNPs, respectively, corresponded to the O–H stretching group of alcohols or phenols as well as water molecules.^39,40^ The strong narrow absorption peaks at 2959, 2920, and 2851 cm^–1^ could be ascribed to the O–H and C–H stretching of carboxylic acid and alkane groups. The peaks at 1639, 1637, and 1635 cm^–1^ could be assigned to the stretching absorption band of the amide carbonyl group (C=O) or amide I, indicating the presence of enzymes or proteins. The absorption peaks at 1384, 1383, and 1379 cm^–1^ could be attributed to the C–H bending of the alkane group and the S=O stretching of the sulfate group. The peaks at 1081, 1080, and 1052 cm^–1^ corresponded to the C–O stretching of the primary alcohol group.^41^ The minor discrepancies in peak positions between AO-SNPs and PDB-SNPs imply the presence of different functional groups involved in SNP biosynthesis, leading to their different sulfur nanoparticle characteristics.

### 3.3 Nematicidal Activity of Cry5B and Cry5B-SNPs against *C. elegans*

**Figure 3:**
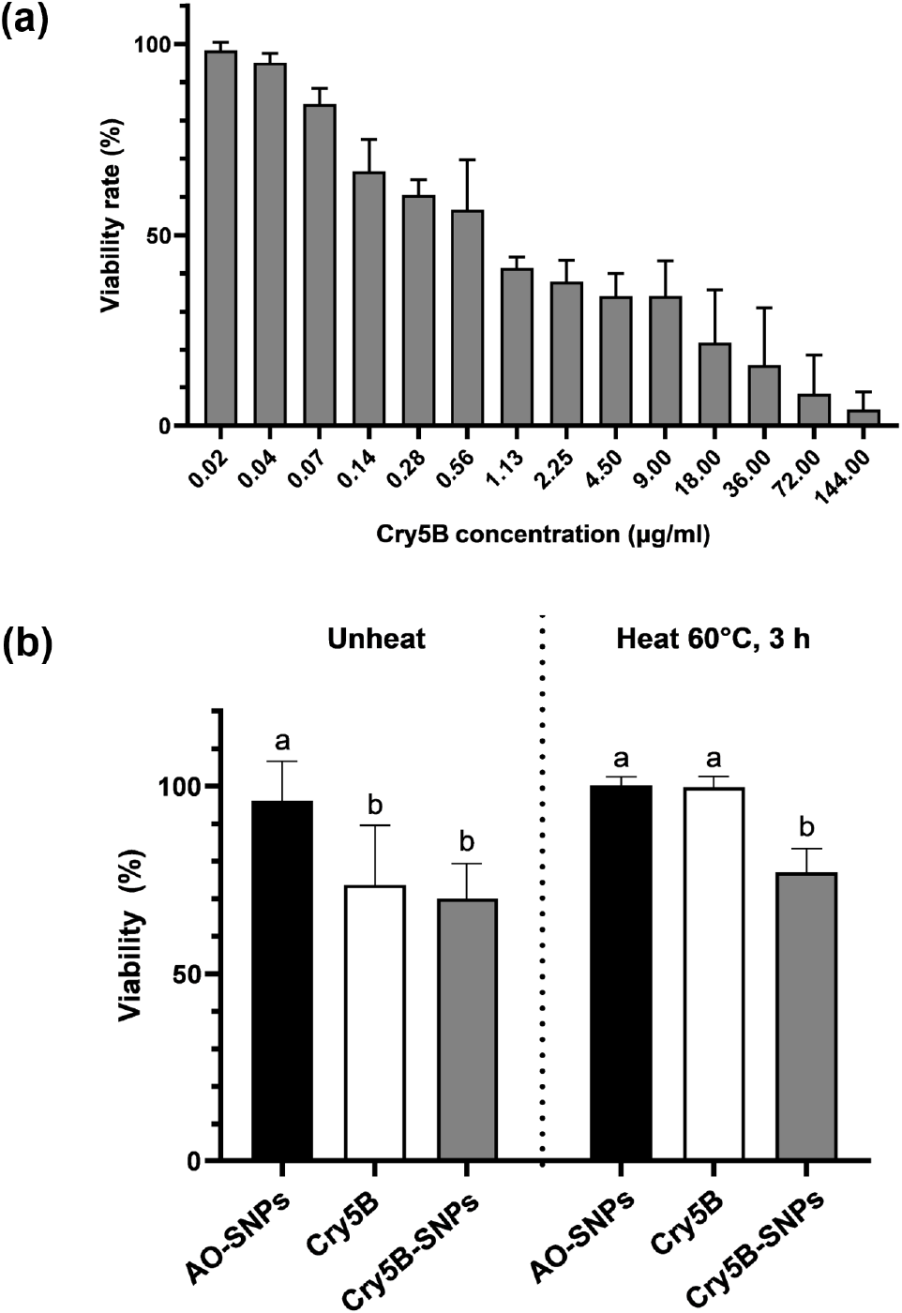
Nematicidal activity of Cry5B and Cry5B-SNPs against *C. elegans* and the thermal stability assay. The viability rate of *C. elegans* after exposure to different concentrations of Cry5B protein for 5 days (a). Nematicidal activity against *C. elegan* of Cry5B-SNPs, Cry5B protein, and AO-SNPs before and after heat exposure at 60 °C for 3 h (b). Different letters indicate significant differences (a: *p*-value > 0.9 (no significance); b: *p*-value < 0.05; two-way ANOVA).

Cry5B protein, expressed as a crystal form with a size of around 140 kDa, was solubilized in 50 mM carbonate buffer pH 10.5 (Figure S2). *C. elegans* that were employed as a model organism were fed with solubilized Cry5B protein at various concentrations ranging from 0.02 to 144 μg/mL, with k-medium serving as a control. Results showed that the Cry5B protein exhibited nematicidal activity against *C. elegans* in a dose-dependent manner (Figure 3a). The LC_50_ value of the Cry5B protein was determined to be 1.21 μg/mL with 95% confidence limits of 0.80–1.83 μg/mL. The LC_90_ value of the Cry5B protein was also calculated to validate the accuracy of the LC_50_ value. The calculated LC_90_ value was 56 μg/mL with 95% confidence limits of 29.1–133.7 μg/mL.

The Cry5B protein was then loaded into/onto AO-SNPs to produce Cry5B-SNPs in order to deliver and prevent degradation of the Cry5B protein when exposed to unfavorable environmental conditions such as heat. However, the loading efficiency of Cry5B-SNPs was only about 14%. This low loading efficiency is likely due to the weak electrostatic interaction between the Cry5B protein and AO-SNPs. This may be because the alkaline buffer used to dissolve the insoluble Cry5B crystal protein makes the protein’s surface negatively charged in solution. Due to the fact that AO-SNPs also have a negative surface charge, loading Cry5B protein into AO-SNPs would result in a high repulsive force and a low loading efficiency. Although it has been reported that Cry5B is highly soluble in a 50 mM citrate (pH 3.0) buffer solution, which would make the protein surface positively charged, the maximum levels of soluble Cry5B protein were observed in a carbonate buffer with a pH of 10.5 in this study. Using this alkaline buffer had no effect on the viability of *C. elegans* as compared to the control; consequently, this buffer was utilized throughout this investigation.

Due to the low loading efficiency in this work, further studies should be performed to optimize the nanoparticle and protein interactions. For example, the buffer and pH for Cry5B protein solubilization may be adjusted to increase the protein solubility and provide an increased surface-positive charge of the Cry5B protein, resulting in stronger electrostatic interactions with the negatively charged AO-SNPs. Furthermore, by introducing cationic substances, such as positively charged amino acids during the nanoparticle synthesis process, it is possible to change the surface charge of AO-SNPs while retaining colloidal stability as previously reported.^42^ To improve the loading efficiency, the ratio of Cry5B protein to AO-SNPs may also be modified.

The nematicidal activity of Cry5B-SNPs against *C. elegans* was assessed in comparison with that of the free Cry5B protein and AO-SNPs. After treating synchronized L4-stage larvae with Cry5B-SNPs, Cry5B protein, and AO-SNPs, alive nematodes were recorded daily under a stereomicroscope for 5 days, and the cumulative percentage of viability was calculated for each sample. Under the unheated condition, the AO-SNPs had the highest percentage of *C. elegans* viability (96.17 ± 10.49%) compared to those of Cry5B-SNPs (70.09 ± 9.34%) and Cry5B protein (73.79 ± 15.82%) (*p*-value < 0.05). The comparable nematicidal activity of Cry5B and Cry5B-SNPs, when used at the same protein concentration, indicated that the *A. oligospora* fungal filtrate played no role in the nematicidal activity but only served as reducing and capping agents for sulfur nanoparticle synthesis. Cry5B was primarily responsible for the nematicidal activity of Cry5B-SNPs against *C. elegans*, and Cry5B retained its nematicidal activity when loaded into/onto AO-SNPs (Figure 3b).

### 3.4 Thermal Stability Analysis of Cry5B and Cry5B-SNPs

It has been reported that the melting temperatures (*T*_m_) of *B. thuringiensis* δ-endotoxins range between 55 and 60 °C.^43^ This study, thus, investigated the Cry5B protein thermal stability upon heat exposure at 60 °C for 3 h among all three treatments, including free Cry5B, free AO-SNPs, and Cry5B-SNPs to ensure that the Cry5B protein undergoes denaturation or unfolding. Results revealed that Cry5B almost completely lost its nematicidal activity after exposure to heat at 60 °C for 3 h, showing the percentage of *C. elegans* viability at 99.85 ± 2.73%. Cry5B-SNPs, on the other hand, retained nematicidal activity by maintaining *C. elegans* viability at 77.11 ± 6.27%, indicating that AO-SNPs could preserve the Cry5B protein’s stability after 3 h of exposure to heat (Figure 3b).

### 3.5 Nematode Pathogenic Analysis of Cry5B and Cry5B-SNPs

**Figure 4:**
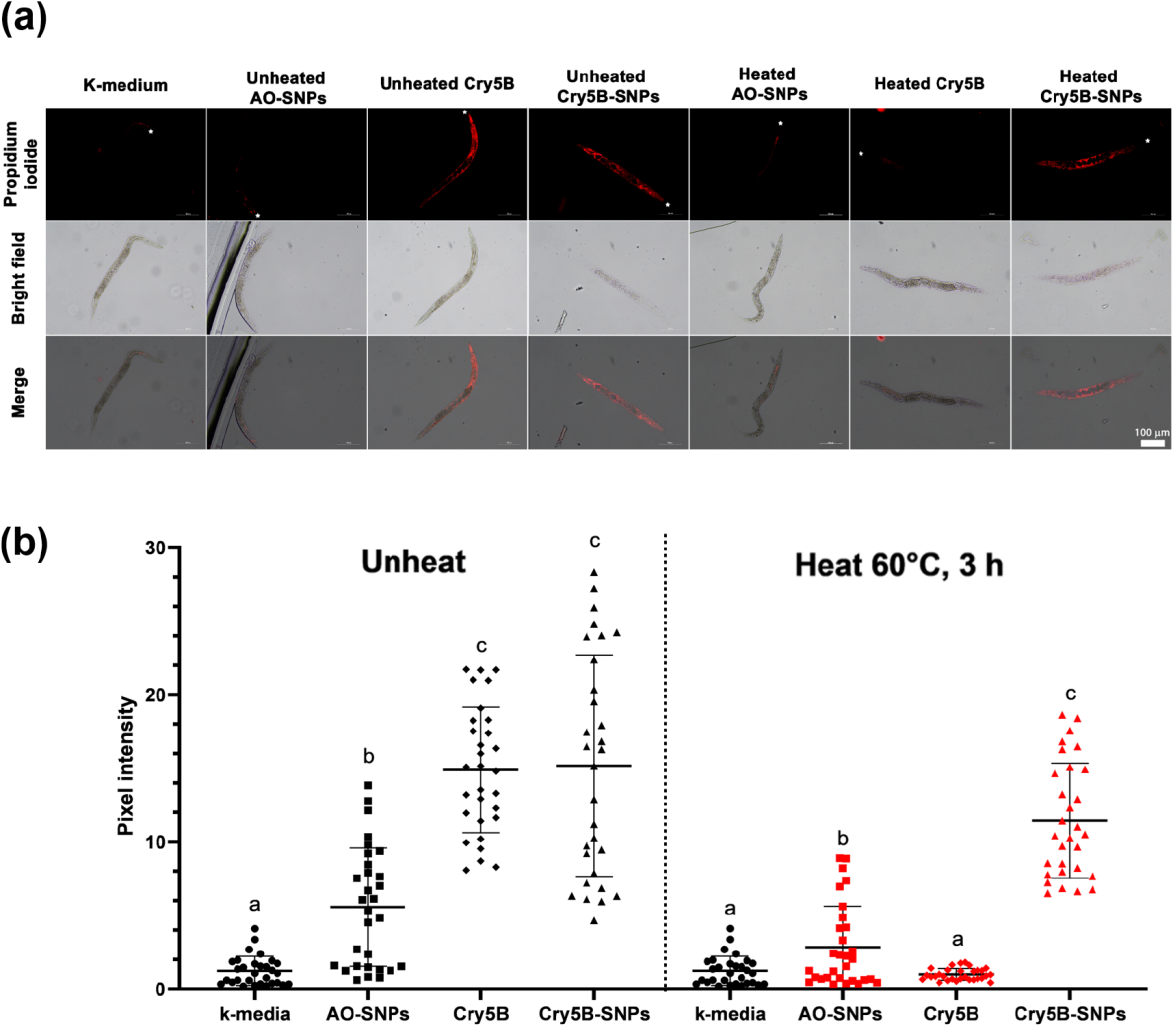
Pathogenic analysis of *C. elegans* exposed to different treatments using a propidium iodide (PI) staining assay. Fluorescence microscope images of *C. elegans* exposed to different treatments, followed by staining with PI (a). Star indicates head. The scale bar indicates 100 μm. Nematode images were acquired under 10× magnification using the G-2A filter. Quantification of PI fluorescence intensity in *C. elegans* cells after fed with either heated or unheated samples of Cry5B, Cry5B-SNPs, and AO-SNPs compared to the control (k-medium) (b). Data are displayed as means ± SD. Each dot indicates each nematode (*n* = 30). Different alphabets indicate significant differences (a: *p*-value > 0.9 (no significance); b: *p*-value < 0.0005; c: *p*-value < 0.0001; two-way ANOVA).

To determine whether Cry5B-SNPs could induce membrane damage via pore formation, we compared the fluorescence intensity of propidium iodide (PI) in *C. elegans* cells after being exposed to either heated or unheated samples of Cry5B, AO-SNPs, and Cry5B-SNPs. PI is an impermeable membrane dye that can penetrate *C. elegans* cells through pores formed by pore-forming toxins.^44,45^ Fluorescence images revealed that *C. elegans* fed unheated Cry5B and Cry5B-SNPs exhibited a loss of cell membrane integrity, as evidenced by the uptake of PI into the cells, whereas nematodes exposed to unheated AO-SNPs displayed a much lower fluorescence intensity. As a control, *C. elegans* fed with the k-medium exhibited no loss of cell membrane integrity (Figure 4a). In addition, the fluorescence intensity was observed throughout the entire body of *C. elegans* (Figure 4b), not only in the digestive tract as previously reported.^46^ Different durations of Cry5B exposure may have contributed to the inconsistent results. This study incubated *C. elegans* with Cry5B protein for 5 days, the same condition as the nematicidal assay, prior to PI staining, whereas a previous study incubated *C. elegans* with Cry5B protein for 7 h prior to PI staining. Necrotic cells are usually permeable for PI, which intercalates in nuclear DNA.^47^ Therefore, we hypothesized that Cry5B could elicit necrotic cell death in nematodes following prolonged exposure to the protein. Nonetheless, the detailed pathogenic mechanism of *C. elegans* treated with Cry5B and Cry5B-SNPs needs to be investigated further.

After 3 h of heat exposure at 60 °C, Cry5B failed to exert the pore-forming activity, as evidenced by the inability of *C. elegans* cells treated with heated Cry5B to uptake PI. Cry5B-SNPs, on the other hand, retained the pore-forming activity after heat exposure at 60 °C by exhibiting comparable fluorescence intensity of PI to unheated Cry5B-SNPs (Figure 4b). Although the release of Cry5B loaded into AO-SNPs was not evaluated in this study, the retained nematicidal and pore-forming activities of Cry5B-SNPs after 3 h of heat treatment at 60 °C suggested that the AO-SNPs would prevent the release of Cry5B. It is, thus, anticipated that when the Cry5B-SNPs were incubated with *C. elegans*, the Cry5B-SNPs, not only the released Cry5B, were ingested by *C. elegans* and could transport Cry5B to *C. elegans* intestinal cells while also inhibiting heat-induced Cry5B protein damage. Although the size of AO-SNPs in this study is relatively bigger than the optimal size of nanoparticles (less than 100 nm), they are sufficiently small to be ingested by *C. elegans*, as an average size of *C. elegans* mouth is in a range of 1–3 μm with 6 symmetrical lips that form a circular cavity to deliver the food to the feeding organ.^48^ Moreover, a previous study showed that the mesoporous silicon particles, which were conjugated with a fluorescent dye, with the size of about 0.4 μm could be directly ingested by the L4-stage *C. elegans* as observed by the distribution of fluorescent signal inside the *C. elegans* gut.^34^ In agreement with the findings of this study, the 0.4 μm particle formulation appeared to be efficiently ingested by L4-stage *C. elegans*. In addition, the loading efficiency of Cry5B protein into mesoporous silicon particles in previous work was about 10%^34^, compared to 14% in this study, indicating that despite the apparent low loading efficiency, Cry5B retained nematicidal activity against *C. elegans* after loading into the nanoparticles in both studies. Taken together, these results indicate that Cry5B-SNPs have the potential to be used to control nematodes, particularly PPNs.

## 4 Conclusions

In this study, we report a simple and low-cost approach for producing sulfur nanoparticles using *A. oligospora* fungal filtrate (AO-SNPs). Biosynthesized AO-SNPs had good stability and uniform spherical shape with a porous structure, making them beneficial for protein loading and preventing protein degradation. After being loaded with the nematicidal Cry5B protein, thermal stability and pathogenic effects of Cry5B-SNPs were assessed in comparison to free Cry5B. Following a 3 h heat treatment at 60 °C, Cry5B-SNPs exhibited greater nematicidal activity in comparison to free Cry5B protein. These findings suggest that the Cry5B-SNP formulation is effective in protecting the Cry5B protein against heat-induced damage and offers potential for future application as a crop protection agent against PPNs. Further investigation is necessary to assess the nematicidal effectiveness of Cry5B-SNPs against root-knot nematodes (*M. incognita*), their shelf life and half-life at room temperature or other temperatures mimicking the field application, as well as their phytotoxic and other long-term effects on the ecosystem.

## Supporting Information Available

FTIR analysis of biosynthesized SNPs using fungal filtrate (AO-SNPs) compared with synthesized SNPs using potato dextrose broth (PBD-SNPs) and SDS-PAGE analysis of Cry5B protein solubilization (PDF)
