# Supplementary material for: Biosynthesis of Cry5B-Loaded Sulfur Nanoparticles using Arthrobotrys oligospora Filtrate: Effects on Nematicidal Activity, Thermal Stability, and Pathogenicity against Caenorhabditis elegans
Source: ACS Omega. 2024 Feb 3;9(6):6945–54. doi: 10.1021/acsomega.3c08653 (PMC10870406; doi:10.1021/acsomega.3c08653)
Supplement: Supplementary file 1 — ao3c08653_si_001.pdf [file ao3c08653_si_001.pdf]

Biosynthesis of Cry5B loaded-sulfur nanoparticles using *Arthrobotrys oligospora* filtrate: Effects on nematicidal activity, thermal stability, and pathogenicity against *Caenorhabditis elegans*

Pasin Jammor[1], Tanatcha Sanguanphun[2], Krai Meemon[2], Boonhiang Promdonkoy[3], Panadda Boonserm[1*]

[1]Institute of Molecular Biosciences, Mahidol University, Salaya, Phuttamonthon, Nakhon Pathom, 73170, Thailand

[2]Department of Anatomy, Faculty of Science, Mahidol University, Rama VI Road, Bangkok, 10400, Thailand

[3]National Center for Genetic Engineering and Biotechnology, National Science and Technology Development Agency, 113 Phahonyothin Road, Khlong Luang, Pathumthani, 12120, Thailand

Corresponding Author

Panadda Boonserm − Email: panadda.boo@mahidol.ac.th

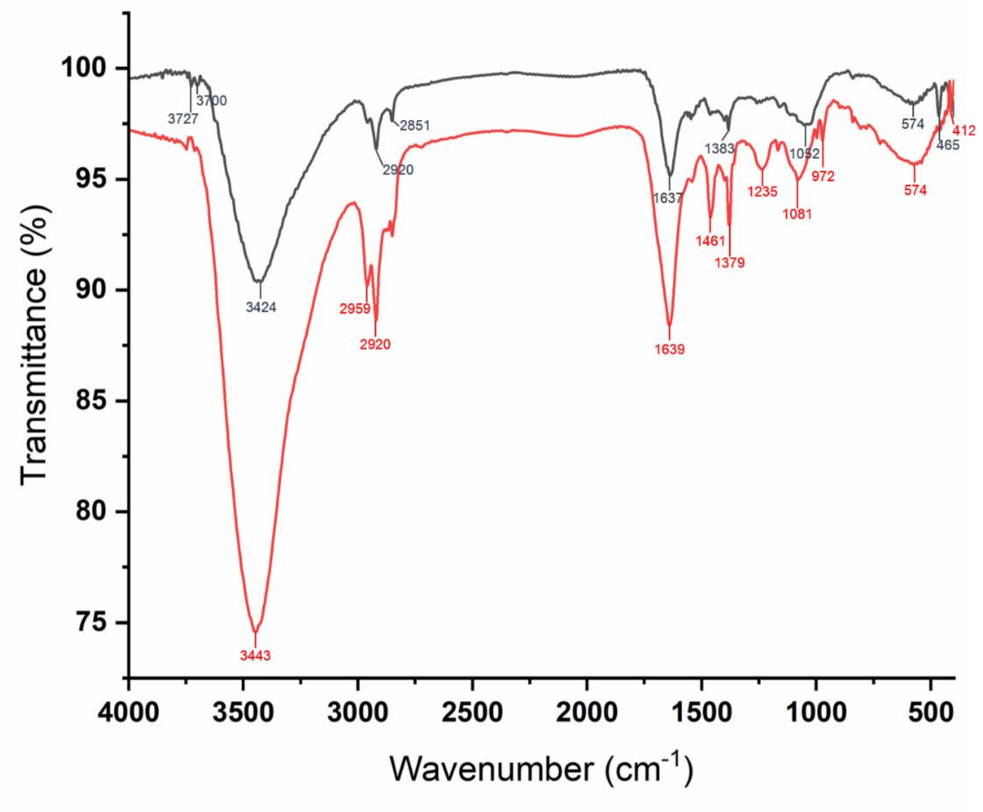

Supplementary Fig. S1 FTIR analysis of biosynthesized SNPs using fungal filtrate (AO-SNPs) (black line) compared with synthesized SNPs using potato dextrose broth (PBD-SNPs) (red line).

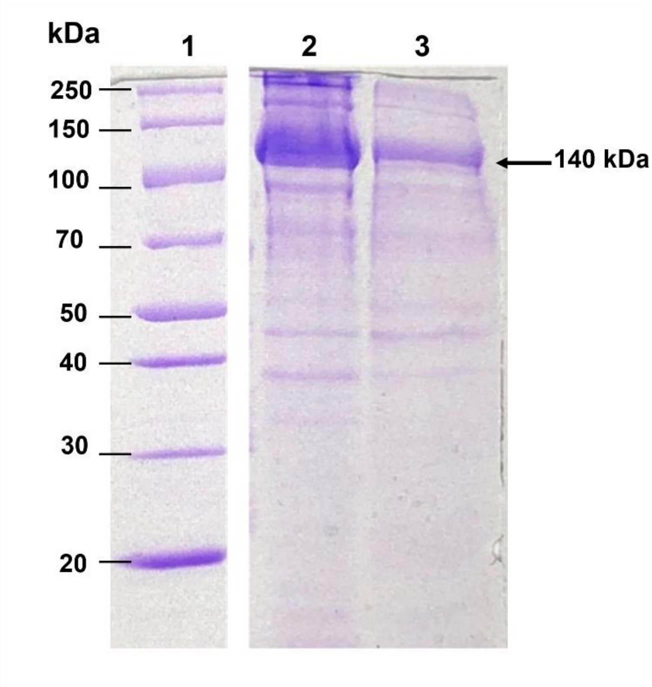

Supplementary Fig. S2 12 % SDS-PAGE analysis of Cry5B protein solubilization. Cry5B protein expressed as a crystal form with a size of around 140 kDa was solubilized in 50 mM carbonate buffer pH 10.5. After centrifugation, the insoluble (lane 2) and soluble (lane 3) fractions were separated and subjected to SDS-PAGE analysis. Lane 1 represents an unstained protein molecular weight marker.
